# Key concepts, methods, findings, and questions about traumatic memories

**DOI:** 10.1002/jts.23164

**Published:** 2025-05-06

**Authors:** Chris R. Brewin

**Affiliations:** ^1^ Research Department of Clinical Educational and Health Psychology University College London London UK

## Abstract

This article is based on a Lifetime Achievement Award lecture delivered at the 40th Annual Meeting of the International Society for Traumatic Stress Studies in Boston (Massachusetts, United States) in September 2024. Understanding traumatic memory involves integrating clinical observations with a wide range of knowledge from philosophy, cognitive and social psychology, and neuroscience. I present definitions of traumatic memory; distinguish voluntary from involuntary forms, such as flashbacks; and introduce relevant concepts that can situate the clinical symptom within a wider framework. The distinction between flashbacks and standard episodic memory has important implications, and I discuss how the methods used to study traumatic memory can preserve it. Using this same perspective, I then review emerging evidence concerning the nature, neural underpinnings, and origin of traumatic memories. The final section reviews some significant unanswered questions for the future. These include the impact of traumatic memories on the experience of self and the implications of delayed onsets for postulating a family of posttraumatic stress disorders with different underlying mechanisms.

Traumatic memories are often considered the essential element of posttraumatic stress disorder (PTSD). Reexperiencing trauma memories predicts the course of the disorder over and above the effects of initial symptom levels (Kleim et al., 2007; Michael et al., 2005) and decreases with successful treatment (Hackmann et al., 2004; Speckens et al., 2006). Specifically addressing flashbacks in therapy appears to contribute to better outcomes (Nijdam et al., 2013). However, despite its central role in maintenance and therapy, traumatic memory is far from being well understood. This article introduces key concepts and methodological issues, reviews major findings, identifies important questions for the future, and summarizes current knowledge.

## CONCEPTS

### Terminology

Typically for clinicians, a traumatic memory refers to one's memory of an event that has traumatized a person (e.g., brought about a disorder such as PTSD). Memories of such events can come to mind involuntarily or be deliberately recalled; both modes of retrieval may also occur simultaneously. However, some psychologists, usually those who are not clinically trained, have used the concept of traumatic memory in the sense of memories related to a potentially traumatic event regardless of whether the person has been traumatized (Shobe & Kihlstrom, 1997; Taylor et al., 2022). This difference can lead to major misunderstandings— for example, that clinical theories make predictions about memories of traumatic events in otherwise healthy people (Brewin & Field, 2024).

There is also a lack of clarity about the differences between involuntary memories, intrusive memories, and flashbacks (Kvavilashvili, 2014). Involuntary autobiographical memories are ordinary memories, typically referred to by cognitive psychologists as *episodic memories*, of past events that pop into the mind unexpectedly without any explicit attempt at recall. Intrusive memories are repeated involuntary memories, usually of a single distressing event or a set of such events—they characterize many disorders, including depression (Brewin et al., 2010), where they may form part of a ruminative process (Pearson et al., 2008).

Flashbacks are a specific type of intrusive memory and constitute a normal response to traumatic events. For example, nurses working in a hospital emergency department may experience flashbacks after difficult or failed resuscitations (Kleim et al., 2015). Typically, they only last for a few days but, if persistent, they may be associated with psychopathology. Flashbacks are sometimes encountered in other disorders (e.g., when there is a trauma history), but primarily characterize PTSD (Bryant et al., 2011).

Flashbacks can be distinguished from other intrusive memories in three main ways: They can only be involuntarily retrieved, they have marked sensory and perceptual characteristics, and they involve some degree of reexperiencing the traumatic event or events in the present. This reexperiencing may involve intense sensations, such as cold or physical pain, that are associated with such events (Macdonald et al., 2018). Clinician identification of flashbacks, therefore, requires an individual to specifically confirm that they reexperience the event as though it was happening again in the present and to describe a corresponding sensory image (typically visual, but sometimes only auditory, olfactory, or kinaesthetic).

Traditionally, the term sometimes has been taken to refer only to very powerful reexperiencing in which the person completely loses contact with their current surroundings. The major diagnostic manuals—the *Diagnostic and Statistical Manual of Mental Disorders* (5th ed.; *DSM‐5*; American Psychiatric Association [APA], 2013) and the *International Statistical Classification of Diseases and Related Health Problems* (11th ed.; *ICD‐11*; World Health Organization [WHO], 2019)—have recently clarified that flashbacks are defined as including a continuum of reexperiencing, with such severe episodes at one extreme and a brief sense of “nowness” at the other. Typically there are a small number of specific scenes, accompanied by marked perceptual details, which may be strung together as though in a film (Ehlers et al., 2004). The characteristics of such memories can be assessed using measures such as the Experiences of Traumatic Memories Questionnaire (Hyland et al., 2024).

The core insight articulated by Pierre Janet in the 19th century and confirmed by clinicians worldwide daily is that memories of traumatic experiences can be dissociated or split off from normal consciousness, resulting in powerful and uncontrollable reenactments of the events (Van der Kolk et al., 1989). Unlike episodic memories, these memories can be triggered by internal or external cues but are not searched for and deliberately retrieved (Brewin, 2014). They are experienced differently than normal memories (Hellawell & Brewin, 2004) and do not correspond to episodic memories, implicit memories, or other types of memories typically studied by cognitive psychologists. In recognizing this distinction, clinical theories differ from episodic memory theories of PTSD (Cohen & Kahana, 2022; Rubin et al., 2008). These theories are based on the idea that PTSD involves very strong memory encoding, which leads to the memories being easily triggered by reminders and persistently intruding. The theories hold that there is no difference in the nature of voluntary and involuntary memories (e.g., flashbacks), only in the method of retrieval. The emphasis on strong encoding means that trauma memories are not expected to be any more disorganized or fragmented than other memories.

### Neural and phenomenological processes

What support might clinical theories of the response to traumatic events find in what is known about sensory systems? Taking the visual system as an exemplar, research confirms that attention may be deliberately controlled (endogenous) or captured automatically by features of the environment (exogenous; Carrasco, 2011). Whereas the former utilizes the slower parvocellular pathway, the latter uses a separate magnocellular pathway designed for the rapid transmission of information. Both pathways project to the visual cortex via the lateral geniculate nucleus of the thalamus. Motion and visual contrast, but not color, capture attention automatically, particularly in situations involving fear or disgust. The magnocellular visual processing system has connections to parts of the brain, such as the amygdala and insula, that are involved in defensive responding.

This same duality is seen in the way visual information is utilized. The slower ventral stream projects from the primary visual cortex to the inferior temporal cortex and is specialized for object recognition and high‐resolution form vision. Objects are represented in an abstract format that permits flexibility, interaction with previously stored information, and an accompanying spatial and temporal context. In contrast, the dorsal stream projects from the primary visual cortex to the superior parietal cortex and motor cortex. It represents visual information from an egocentric perspective and provides a rapid pathway for relatively unprocessed information to influence action, including defensive action.

It appears, therefore, that the brain is equipped with parallel visual pathways that may behave differently in traumatic situations. According to the revised *dual representation theory of PTSD* (R‐DRT; Brewin et al., 2010), these two modes of visual processing lead to different types of representation, contextualized memory (based on ventral stream processing), and sensation‐based memory (based on dorsal stream processing). During a traumatic event, attention tends to be narrowed, and structures involved in the organization and encoding of autobiographical memory, such as the prefrontal cortex and hippocampus, are down‐regulated. Briefly, apprehended sensory information can, nevertheless, be captured in the form of images accompanied by arousal and body state information in sensation‐based memory.

The R‐DRT proposes that, although normally integrated, the extreme stress associated with some traumatic events leads to a weakening of the connections between the two types of memory. Subsequently, reminders of the event can lead to the retrieval of sensation‐based memories that, in the absence of a corresponding contextualized memory, result in specific moments being reexperienced as flashbacks. When the person is safe, the information contained in flashbacks can be re‐presented to consciousness to be integrated into the autobiographical knowledge contained in the contextual memory system.

Involuntary traumatic memories and flashbacks are examples of what philosophers refer to as “prereflective experience.” This pure phenomenal experience is bound to the current situation and relatively independent of voluntary attention. Like sensation‐based memory in R‐DRT, prereflective experience is perceived from an egocentric viewpoint and is difficult to put into words. An individual experiences it but does not think about it and does not integrate it into their knowledge of the self. Apart from Tulving (1985), most cognitive psychologists studying memory have neglected prereflective experience in favor of the explicit/implicit memory distinction. Implicit memory occurs exclusively outside of awareness and, therefore, does not capture the reexperiencing that is part of PTSD.

Many psychiatric symptoms and dissociative experiences, such as flashbacks, mood changes, automatic thoughts, voice hearing, and bodily sensations, have a prereflective quality. It is an important concept that has much to offer psychopathology. The “memory and identity” theory of *ICD‐11* complex PTSD (CPTSD; Hyland et al., 2023) discusses how prereflective experience contributes to both traumatic memory and to the negative identity states that form part of this disorder.

## METHODS OF ENQUIRY

An important measurement issue is how involuntary memories are identified in diagnostic assessments of PTSD. *DSM‐5* Symptom B1 refers to “recurrent, involuntary, and intrusive distressing memories of the traumatic event(s)” (APA, 2013). The manual clarifies that the “emphasis is on recurrent memories of the event that usually include sensory, emotional, or physiological behavioral components.” These features may also be present in intrusive episodic memories, although, in practice, most intrusive traumatic memories will likely consist of flashbacks involving some degree of reliving. More precise measurement of flashbacks requires *DSM‐5* Symptom B3 or a measure specifically tailored to the *ICD‐11*, where reexperiencing explicitly involves a sense of “nowness.” There is some evidence that the reliance of diagnostic interviews and questionnaires on retrospective recall is likely to underestimate the number of flashbacks an individual with PTSD has recently experienced and that an electronic diary would be more accurate (Priebe et al., 2013).

In the context of research, it is important to consider the relevance of the distinction between flashbacks and episodic memories when eliciting the recall of traumatic events. Methods that are based simply on collecting trauma narratives will generate a mixture of both types of memory. Similarly, methods that present participants with trauma reminders and measure their reactions need to determine whether, and at what points, the reminders produced flashbacks. Standard script‐driven imagery procedures (Shin et al., 1999) can likewise lead to a mixture of both voluntary and involuntary recall.

The method developed by Hellawell and Brewin (2002) to address these issues starts with individuals with PTSD completing a written narrative of their traumatic event. Next, they are asked to mark those words and sentences where they experienced a flashback while writing and those (corresponding to ordinary memories) where there was no flashback. The narrative can then be divided into two parts based on the aggregates of “flashback” and “ordinary memory” sections. A variant of this method is to have individuals watch a video recorded while they were producing their narrative and indicate when flashbacks occurred.

Although the concept of a flashback is outside most people's experience, nobody with PTSD we have tested using these methods has failed to understand the task or has reported difficulty with it. The results are invariably highly idiosyncratic in terms of how many flashbacks there are, how long they last, and where in the narrative they occur. This emphasizes that individual‐level analysis is required to assess the two types of memory accurately.

In research conducted to validate these methods, compared to episodic memory sections, flashback sections have been rated as more negative and arousing (Brewin, Huntley, & Whalley, 2012); accompanied by increases in heart rate (Chou et al., 2018); shown to include more sensory words and mentions of death (Hellawell & Brewin, 2004); accompanied by increased autonomic and motor behaviors, such as eye movements, breathing changes, and vocalizations; and more likely to interfere with the writing of the trauma narrative (Hellawell & Brewin, 2002). In one study, participants were asked to write trauma narratives and identify flashback and episodic memory sections (Session 1; Brewin, Huntley, & Whalley, 2012). In Session 2, words and short phrases from the flashback and episodic memory sections of both the participant's trauma narrative and other participants’ narratives, matched on word length and frequency, were presented to participants in a magnetic resonance imaging (MRI) scanner. Participants then performed a simple recognition task in which they were asked to determine whether a word or phrase came from their narrative. The study showed that flashbacks were more likely to occur for phrases rather than words from the person's own narrative. Approximately 54% of phrases that had elicited a flashback in Session 1 did so in Session 2, whereas 32% of phrases that had not previously elicited a flashback did so. This illustrates that the occurrence of flashbacks varies according to the situation and is only partially predictable.

## MAJOR FINDINGS

### Involuntary recall

Intrusive traumatic memories typically consist of worst moments (i.e., “hotspots”; Grey et al., 2001; Hoppe et al., 2022) or, alternatively, moments that warn of impending danger (Ehlers et al., 2002). The experience is distinct from other types of intrusive cognitive processes, such as rumination (Speckens et al., 2007). As predicted by the R‐DRT, flashbacks are more likely to involve emotions such as fear, helplessness, and horror that were experienced during the traumatic event whereas episodic memories are more likely to involve subsequent reflective emotions after the event, such as sadness and guilt (Hellawell & Brewin, 2004). This is an important finding that emphasizes that flashbacks do not just reflect moments of more extreme emotion, as episodic memory theories would suggest, but involve specific, survival‐related emotions.

Although naturalistic data on the accuracy of flashbacks are lacking, some indication can be derived from research such as the study by Brewin, Huntley, and Whalley (2012), which assessed whether words or phrases from an individual's own narrative could be discriminated from those of another person's narrative. In this study, recognition was highest for phrases that had elicited a flashback both in the original narrative and at recall (mean hit rate: .96) and lowest for single words that had not elicited a flashback in either the original narrative or at recall (mean hit rate: .69). Notably, approximately 70% of participants reported at least one flashback to a word or phrase from another participant's (i.e., control) narrative (*M* = 3.8 times). Words and phrases from this control narrative were significantly more likely to be incorrectly judged as the participant's own when they were accompanied by a flashback (71% incorrect) compared to when they were not (19% incorrect). This result accords with evidence that features such as emotion and sensory vividness are often used to help people discriminate between events they have experienced and those they have only imagined (Johnson, 2006).

The data presented by Brewin, Huntley, and Whalley (2012) suggest that flashbacks are generally associated with higher accuracy but that they can sometimes be misleading. The findings may be relevant to traumatic memories from childhood that are recovered after many years, which also tend to return initially in the form of flashbacks (Andrews et al., 2000). It is possible that in a minority of instances, an accompanying flashback may persuade the individual that their experience corresponds to a real event when this is, in fact, untrue.

Responses to specific words and phrases during a subsequent test can be used to discriminate neural responses to flashbacks and episodic trauma memories. Data from the only imaging study to use these methods (Whalley et al., 2013) suggest that consistent with the R‐DRT, flashbacks are associated with widespread activation in the dorsal visual stream and in areas associated with egocentric representation and the involuntary capture of attention. Reduced activation in the parahippocampal area of the ventral visual stream was also noted. A possible interpretation is that flashbacks do not involve normal recollection but are more similar to an extreme form of familiarity response.

The extreme heterogeneity of PTSD (Bryant et al., 2023) suggests that biological studies might fruitfully investigate how specific symptoms, like intrusive memories and flashbacks, correlate with biomarkers rather than compare groups based on their overall diagnosis. Initial studies have found that these intrusive symptoms appear to account for most of the variance in biomarkers as varied as brain volume (Kroes et al., 2011) and gene expression (Rusch et al., 2019).

There has been considerable interest in whether mental states such as dissociation during a traumatic event are related to the later development of PTSD. Research can go further to ask whether the specific moments of a traumatic event that come back repeatedly as flashbacks and intrusive memories are associated with different peritraumatic states in comparison to the moments that do not later intrude. This can be addressed by directly comparing, within the same individual, the recall of such states during the moments that feature in a specific intrusive memory with recall of the same states during moments that feature in a matched nonintrusive memory. States during intrusive memory moments can also be compared with states during the moments that make up a very distressing memory in individuals who do not report intrusions.

This design was adopted to study correlates of intrusive memory development in survivors of a major earthquake in Central Italy. Because there are numerous dissociation scales with overlapping content, the first step was to conduct exploratory structural equation modeling on 63 items from six common peritraumatic response scales completed by 308 earthquake survivors (Massazza, Joffe, Hyland, & Brewin, 2021). The best model fit was a five‐factor solution consisting of mental defeat (e.g., “In my mind, I gave up”), somatoform dissociation (e.g., “It felt as if my body, or parts of it, disappeared”), cognitive overload (e.g., “I did not understand what was going on”), immobility (e.g., “I felt temporarily paralyzed or stiff”), and distress (e.g., “I thought I might die”).

In a subsequent analysis (Massazza, Joffe, & Brewin, 2021), all peritraumatic reactions except mental defeat were reported to be much higher during moments that came back as intrusions when compared to both nonintrusive moments in the same participants and “most distressing” memories in participants who did not report any intrusions. Intrusive memories were also characterized by higher levels of fear, anxiety, and helplessness, all emotions associated with the original traumatic event rather than subsequent reflection. These results support the dual representation theory and replicate other findings linking intrusive trauma memories specifically to fear rather than other emotions (Hellawell & Brewin, 2004; Reynolds & Brewin, 1999).

### Voluntary recall

There has been marked disagreement about whether voluntarily produced trauma memories or narratives provided by individuals with PTSD are sometimes disorganized or incoherent, as some clinical theories have proposed (Brewin & Ehlers, 2024). Although disordered recall in the form of significant gaps in memory is noted as a symptom of PTSD within the *DSM‐5* (APA, 2013), some psychologists have argued that traumatic memories are normally clear and strong (McNally, 2003; Rubin et al., 2008). These same psychologists have been skeptical about the validity of many recovered memories of trauma, claiming that as traumatic events are strongly encoded, they are unlikely to be forgotten. The presence of disorganization or incoherence undermines this claim by showing that trauma memories are not invariably clear and strong.

Some scholars have also suggested that clinical theories about memory incoherence imply that the trauma memory is not integrated into the life story (Berntsen et al., 2003). This is a misunderstanding. Clinical theories describe the characteristics of episodic memories of the event, not the representation of the event in semantic memory. It is perfectly possible for memories of a specific event (i.e., episodic memory) to be disjointed while the event looms large and is highly significant in one's personal history. Indeed, leading trauma theorists have frequently argued that such events form a central reference point because they tend to contradict previous assumptions (Horowitz, 1976; Janoff‐Bulman, 1992).

A number of studies have investigated memory incoherence or disorganization using clinically based methods for eliciting trauma narratives in reliving sessions: Participants are given specific instructions to imagine the events are happening again, to keep their eyes closed, and to describe the event in as much detail as possible (e.g., Harvey & Bryant, 1999). The main measures are then derived by segmenting the narrative into individual utterances, which are rated by independent judges for fragmentation (repetitions, unfinished thoughts, speech fillers) and disorganization (utterances that imply confusion or disjointed thinking; Foa et al., 1995). In addition, clinical investigators have rated narratives globally for disorganization or have had participants themselves do this.

Other researchers have used similar global ratings but have introduced measures worded in the opposite direction (higher scores indicating organization or coherence), assuming that these assess the same qualities albeit inversely (e.g., O'Kearney et al., 2007). For example, instead of participants rating their narrative with an item like “My memory comes in pieces with missing bits,” they rate the memory with an item like “While remembering the event, it comes to me in words or in pictures as a coherent story or episode and not as an isolated fact, observation, or scene.” Other investigators have used unvalidated computer‐scored measures to assess such constructs as word concreteness, referential cohesion, and temporal connectives. The appropriateness of these methods has been criticized (Gray & Lombardo, 2001) and they are not considered further here.

An initial review (Brewin, 2014) concluded that studies using the clinically based measures described by Foa et al. (1995) have reliably demonstrated evidence of the disorganization and fragmentation of trauma memories. However, this was challenged (Rubin, Deffler, et al., 2016) on the basis of negative results obtained from a number of studies using global ratings of organization and disorganization combined. These investigators reported no significant differences between participants with and without PTSD in their studies or those reported by other researchers using similar methods. In response, I proposed that this could be due to the differences in methods used in their studies, which did not find that PTSD was related to disorganization, and the clinical studies, which did routinely find such an association (Brewin, 2016). This proposal was dismissed as being “without merit” and “unsupported by data,” fitting “a pattern of selective omissions” and consisting of “outdated beliefs” (Rubin, Berntsen, et al., 2016).

We (Brewin & Field, 2024) responded to these challenges by conducting a meta‐analysis using multilevel methods that allowed for different types of dependent measures from the same study to be analyzed separately. Four types of measure were distinguished: detailed utterance ratings of disorganization or incoherence from the clinical studies using Foa et al.’s (1995) methods, global ratings of disorganization and incoherence from the same set of studies (self‐report or judge ratings), global ratings of disorganization and incoherence from the studies that did not use Foa et al.’s methods, and global ratings of organization and coherence from these same studies. In the overall model, there was a strong positive association between disorganization in the trauma narrative and PTSD. Both effect sizes derived from the studies using Foa et al.’s methods were large and significant, as was the effect size derived from the studies that did not use these methods, in which high scores on the global ratings indicated more disorganization. In contrast, when high ratings indicated organization or coherence in these same studies, the effect was close to 0 and nonsignificant.

The analyses are clear that measures that directly address fragmentation or incoherence, whatever their nature, reliably showed higher scores for samples with PTSD. Measures addressing their opposite—coherence—did not show these effects. This moderation effect was significant and demonstrable even within the small subset of six studies conducted by the primary critics of clinical theories, Rubin, Bernsten, and colleagues (2016). The data demonstrate that a trauma narrative can have overall elements of coherence—for example, it can be situated in context and have a logical structure—but at the same time may show signs of incoherence or fragmentation. Like measures of positive and negative social support (Andrews et al., 2003), measures of coherence and incoherence are not logical opposites and have different implications.

## QUESTIONS

There are many outstanding questions about intrusive trauma memories and flashbacks. For example, if they depend on the magnocellular pathway and dorsal visual stream, which respond more to motion and larger objects, why are the images people reexperience so detailed and colorful? One proposal is that a strong emotional event can rapidly activate endogenous mechanisms of plasticity in the hippocampus and amygdala for a short period (Diamond et al., 2007). Then, the induction of new plasticity is suppressed in both structures, which facilitates the memory consolidation process. Diamond et al. suggest that this results in the hippocampus storing disembodied fragments of an experience that lack the normal depth of processing of context. The implication is that the content of flashbacks depends not on the complete dominance of the dorsal stream but on time‐dependent interactions between the visual streams.

Another question concerns the possible impact of intrusive trauma memories on individuals’ sense of themselves. There is evidence that in PTSD, intrusions lead individuals to draw negative conclusions about their own characteristics (Dunmore et al., 1999; Ehlers & Steil, 1995). One study found that specifically, the perceived nowness of an intrusion was related to experiencing more fear, helplessness, guilt, and shame (Kleim et al., 2013). More recently, higher scores on a measure of intrusive memories were found to be associated with the negative self‐concept symptoms of *ICD‐11* CPTSD (Hyland et al., 2024).

Other intrusions that may accompany PTSD, such as hearing voices (McCarthy‐Jones & Longden, 2015), tend to trigger negative feelings about the self (Brewin & Patel, 2010). Voices are often dominant and critical, and the individual has a submissive relationship with them. Intrusive memories may also trigger negative emotions and experiences connected with previous relationships. There is a great deal of scope for qualitative studies to investigate in more depth the impact of intrusions and what qualities or types of content are the most difficult to manage. These could build on the observations of Ehlers and Steil (1995), extending them from the self‐concept to the subjective sense of self. Hyland et al. (2023) proposed that the self in *ICD*‐*11* CPTSD is experienced as worthless, alienated from others, and fragmented or even nonexistent. Some of these reactions are likely to be prompted or exacerbated by intrusive memories.

Further questions are related to the observation that memories can sometimes acquire traumatic characteristics over time, which contradicts the common view of trauma as invariably being overwhelming from the outset. This may occur in delayed‐onset PTSD, for instance. In one of the few studies to have investigated this issue, military veterans with delayed‐ and immediate‐onset PTSD reported similar symptoms and amounts of trauma exposure but could be distinguished by their emotional and dissociative reactions to their index traumatic event (Andrews et al., 2009). Immediate onset was associated with strong acute reactions, suggesting the person was overwhelmed by the event, as well as with persisting dissociation. Delayed onset was associated with a normal acute response to trauma, with the eventual onset triggered by general rather than traumatic stressors. Delayed onset could be separately predicted by a poor disciplinary record prior to trauma exposure (Brewin, Andrews, et al., 2012), suggesting the presence of additional risk factors.

Also described are reactivations of traumatic memories, particularly in the elderly (Hiskey et al., 2008a). Indications are that intrusive memories lose none of their power, even after many years (Hiskey et al., 2008b). There are several theoretical reasons why traumatic memories may only start to intrude sometime after a traumatic event. One is that events change in their emotional significance. Additional information may change the perception of the traumatic stressor, resulting in its being seen in a more threatening light. This process has been called “stimulus revaluation” (Davey, 1989). Another potential reason is that there is an increase in retrieval cues. For example, military veterans sometimes report an increase in intrusive memories connected with anniversaries or memorial events. In addition, various factors, such as age or cognitive decline, may make it harder to suppress unwanted intrusive memories (Mota et al., 2016). All these mechanisms await systematic research. At this stage of knowledge, detailed case studies or retrospective interview studies, supplemented by cognitive assessments and informant interviews, are likely to be the most informative approaches.

## SUMMARY

There is a wealth of evidence that high levels of emotion are associated with both memory enhancement and memory impairment. Although most of these changes have been demonstrated in clinical populations, such as individuals with PTSD, recent laboratory research has found that even low levels of negative affect have a selective effect in healthy participants, leading to better memory for a central item but weaker memory for its surrounding context (Bisby & Burgess, 2017).

Consistent with theories that emphasize how stress selectively affects certain brain structures, such as the hippocampus, and certain types of learning (Jacobs & Nadel, 1985), animal studies have found that high levels of stress facilitate Pavlovian conditioning but impair hippocampal‐dependent forms of memory, such as relational and explicit learning (Kim & Diamond, 2002; Sandi & Pinelo‐Nava, 2007). In one study, firefighters experiencing the highest levels of stress demonstrated the greatest recall impairment for incidents they attended (Metcalfe et al., 2019), and another study showed that special forces undergoing the most stressful form of interrogation had the poorest eyewitness memory of their interrogator (Morgan et al., 2004).

These findings support the repeated clinical observations, reviewed in this article, of disorganized or fragmented voluntary recall of traumatic events associated with PTSD. Appreciation of the real nature of trauma memory is often crucial in legal settings, where there may be an incorrect expectation on the part of juries that memories are invariably lucid, detailed, and complete. The findings also support the well‐documented instances of forgetting and subsequently recovering traumatic memories (Andrews et al., 1999; Dalenberg, 2006; DePrince et al., 2012). Detailed case examples of recovered traumatic memory, as well as additional materials (e.g., relevant scientific articles), are provided in the Recovery Memory Archive (n.d.), which is maintained by Dr. Ross Cheit and his team. It is also important to note that recovered memories may be erroneous and the product of suggestion (Lindsay & Read, 1995) or source monitoring errors (Johnson, 2006).

In PTSD, memory facilitation is mainly evident in the repeated intrusion of involuntary memories in the form of flashbacks of varying severity. Most of these intrusions appear to be prereflective, composed of images or snapshots from a few moments during the traumatic event, and triggered by internal or external cues. Consistent with their highly sensory nature, “nowness,” and absence of context, there is preliminary evidence that they depend at least in part on a nonhippocampal form of memory. This form of memory is thought to become dominant during brief moments when individuals experience extreme fear, reacting with somatoform dissociation, cognitive overload, or immobility.

Research on the delayed onset and reactivation of PTSD (Andrews et al., 2009; Sachs‐Ericsson et al., 2016) has suggested that increased levels of subsequent stress might make it hard to continue to inhibit trauma memories. The implication is that some cases of PTSD represent a causal pathway that differs from the usual response to an overwhelming initial event. Rather, a person who is innately vulnerable to stress or has endured high levels of stressor exposure accumulates an unsustainable burden over time, resulting in the delayed onset of symptoms. This may be more common among military, police, and other emergency services personnel because of the nature of their work. These observations add to evidence from the statistical independence of *ICD‐11* PTSD and CPTSD (Redican et al., 2021) and the dissociative subtype of *DSM‐5* PTSD (Wolf et al., 2023) that it may be time to think not so much about a single disorder as about a family of posttraumatic stress disorders in which traumatic memories play a slightly different role.

## References

[jts23164-bib-0001] American Psychiatric Association . (2013). Diagnostic and statistical manual of mental disorders (5th ed.). 10.1176/appi.books.9780890425596

[jts23164-bib-0002] Andrews, B. , Brewin, C. R. , Ochera, J. , Morton, J. , Bekerian, D. A. , Davies, G. M. , & Mollon, P. (1999). Characteristics, context and consequences of memory recovery among adults in therapy. British Journal of Psychiatry, 175, 141–146. 10.1192/bjp.175.2.141 10627796

[jts23164-bib-0003] Andrews, B. , Brewin, C. R. , Ochera, J. , Morton, J. , Bekerian, D. A. , Davies, G. M. , & Mollon, P. (2000). The timing, triggers and qualities of recovered memories in therapy. British Journal of Clinical Psychology, 39, 11–26. 10.1348/014466500163077 10789025

[jts23164-bib-0004] Andrews, B. , Brewin, C. R. , & Rose, S. (2003). Gender, social support, and PTSD in victims of violent crime. Journal of Traumatic Stress, 16(4), 421–427. 10.1023/a:1024478305142 12895025

[jts23164-bib-0005] Andrews, B. , Brewin, C. R. , Stewart, L. , Philpott, R. , & Hejdenberg, J. (2009). Comparison of immediate‐onset and delayed‐onset posttraumatic stress disorder in military veterans. Journal of Abnormal Psychology, 118(4), 767–777. 10.1037/a0017203 19899846

[jts23164-bib-0006] Berntsen, D. , Willert, M. , & Rubin, D. C. (2003). Splintered memories or vivid landmarks? Qualities and organization of traumatic memories with and without PTSD. Applied Cognitive Psychology, 17(6), 675–693. 10.1002/acp.894

[jts23164-bib-0007] Bisby, J. , & Burgess, N. (2017). Differential effects of negative emotion on memory for items and associations, and their relationship to intrusive imagery. Current Opinion in Behavioral Sciences, 17, 124–132. 10.1016/j.cobeha.2017.07.012 29238740 PMC5719982

[jts23164-bib-0008] Brewin, C. R. (2014). Episodic memory, perceptual memory, and their interaction: Foundations for a theory of posttraumatic stress disorder. Psychological Bulletin, 140(1), 69–97. 10.1037/a0033722 23914721

[jts23164-bib-0084] Brewin, C. R. (2016). Coherence, disorganization, and fragmentation in traumatic memory reconsidered: A response to Rubin et al. (2016). Journal of Abnormal Psychology, 125(7), 1011–1017. 10.1037/abn0000154 27732029

[jts23164-bib-0009] Brewin, C. R. , Andrews, B. , Hejdenberg, J. , & Stewart, L. (2012). Objective predictors of delayed‐onset post‐traumatic stress disorder occurring after military discharge. Psychological Medicine, 42(10), 2119–2126. 10.1017/s0033291712000189 22348623

[jts23164-bib-0010] Brewin, C. R. , & Ehlers, A. (2024). Post‐traumatic stress disorder. In M. J. Kahana & A. D. Wagner (Eds.), The Oxford handbook of human memory: Foundations and applications (Vol. 2, pp. 1945–1971). Oxford University Press.

[jts23164-bib-0011] Brewin, C. R. , & Field, A. P. (2024). Meta‐analysis shows trauma memories in posttraumatic stress disorder lack coherence: A response to Taylor et al. (2022). Clinical Psychological Science, 12(5), 1027–1033. 10.1177/21677026241240456

[jts23164-bib-0012] Brewin, C. R. , Gregory, J. D. , Lipton, M. , & Burgess, N. (2010). Intrusive images in psychological disorders: Characteristics, neural mechanisms, and treatment implications. Psychological Review, 117(1), 210–232. 10.1037/a0018113 20063969 PMC2834572

[jts23164-bib-0013] Brewin, C. R. , Huntley, Z. , & Whalley, M. G. (2012). Source memory errors associated with reports of posttraumatic flashbacks: A proof of concept study. Cognition, 124(2), 234–238. 10.1016/j.cognition.2012.05.002 22658646 PMC3396838

[jts23164-bib-0014] Brewin, C. R. , & Patel, T. (2010). Auditory pseudohallucinations in United Kingdom war veterans and civilians with posttraumatic stress disorder. Journal of Clinical Psychiatry, 71(4), 419–425. 10.4088/JCP.09m05469blu 20361915

[jts23164-bib-0015] Bryant, R. A. , Galatzer‐Levy, I. , & Hadzi‐Pavlovic, D. (2023). The heterogeneity of posttraumatic stress disorder in *DSM‐5* . JAMA Psychiatry, 80(2), 189–191. 10.1001/jamapsychiatry.2022.4092 36477192 PMC9856854

[jts23164-bib-0016] Bryant, R. A. , O'Donnell, M. L. , Creamer, M. , McFarlane, A. C. , & Silove, D. (2011). Posttraumatic intrusive symptoms across psychiatric disorders. Journal of Psychiatric Research, 45(6), 842–847. 10.1016/j.jpsychires.2010.11.012 21159353

[jts23164-bib-0017] Carrasco, M. (2011). Visual attention: The past 25 years. Vision Research, 51(13), 1484–1525. 10.1016/j.visres.2011.04.012 21549742 PMC3390154

[jts23164-bib-0018] Chou, C.‐Y. , La Marca, R. , Steptoe, A. , & Brewin, C. R. (2018). Cardiovascular and psychological responses to voluntary recall of trauma in posttraumatic stress disorder. European Journal of Psychotraumatology, 9(1), Article 1472988. 10.1080/20008198.2018.1472988 29887977 PMC5990938

[jts23164-bib-0019] Cohen, R. T. , & Kahana, M. J. (2022). A memory‐based theory of emotional disorders. Psychological Review, 129(4), 742–776. 10.1037/rev0000334 35389717 PMC9256582

[jts23164-bib-0020] Dalenberg, C. (2006). Recovered memory and the Daubert criteria: Recovered memory as professionally tested, peer reviewed, and accepted in the relevant scientific community. Trauma Violence & Abuse, 7(4), 274–310. 10.1177/1524838006294572 17065548

[jts23164-bib-0021] Davey, G. C. L. (1989). UCS revaluation and conditioning models of acquired fears. Behaviour Research and Therapy, 27(5), 521–528. 10.1016/0005-7967(89)90086-7 2684133

[jts23164-bib-0022] DePrince, A. P. , Brown, L. S. , Cheit, R. E. , Freyd, J. J. , Gold, S. N. , Pezdek, K. , & Quina, K. (2012). Motivated forgetting and misremembering: Perspectives from betrayal trauma theory. In R. F. Belli (Ed.), True and false recovered memories: Toward a reconciliation of the debate (pp. 193–242). Springer. 10.1007/978-1-4614-1195-6_7 22303768

[jts23164-bib-0023] Diamond, D. M. , Campbell, A. M. , Park, C. R. , Halonen, J. , & Zoladz, P. R. (2007). The temporal dynamics model of emotional memory processing: A synthesis on the neurobiological basis of stress‐induced amnesia, flashbulb and traumatic memories, and the Yerkes–Dodson law. Neural Plasticity, 2007, Article 60803. 10.1155/2007/60803 17641736 PMC1906714

[jts23164-bib-0024] Dunmore, E. , Clark, D. M. , & Ehlers, A. (1999). Cognitive factors involved in the onset and maintenance of posttraumatic stress disorder (PTSD) after physical or sexual assault. Behaviour Research and Therapy, 37(9), 809–829. 10.1016/s0005-7967(98)00181-8 10458046

[jts23164-bib-0025] Ehlers, A. , Hackmann, A. , & Michael, T. (2004). Intrusive re‐experiencing in post‐traumatic stress disorder: Phenomenology, theory, and therapy. Memory, 12(4), 403–415. 10.1080/09658210444000025 15487537

[jts23164-bib-0026] Ehlers, A. , Hackmann, A. , Steil, R. , Clohessy, S. , Wenninger, K. , & Winter, H. (2002). The nature of intrusive memories after trauma: The warning signal hypothesis. Behaviour Research and Therapy, 40(9), 995–1002. 10.1016/s0005-7967(01)00077-8 12296496

[jts23164-bib-0027] Ehlers, A. , & Steil, R. (1995). Maintenance of intrusive memories in posttraumatic stress disorder: A cognitive approach. Behavioural and Cognitive Psychotherapy, 23(3), 217–249. 10.1017/S135246580001585X 21241539 PMC2887304

[jts23164-bib-0028] Foa, E. B. , Molnar, C. , & Cashman, L. (1995). Change in rape narratives during exposure therapy for posttraumatic stress disorder. Journal of Traumatic Stress, 8(4), 675–690. 10.1007/bf02102894 8564278

[jts23164-bib-0029] Gray, M. J. , & Lombardo, T. W. (2001). Complexity of trauma narratives as an index of fragmented memory in PTSD: A critical analysis. Applied Cognitive Psychology, 15(7), S171–S186. 10.1002/acp.840

[jts23164-bib-0030] Grey, N. , Holmes, E. , & Brewin, C. R. (2001). Peritraumatic emotional “hot spots” in memory. Behavioural and Cognitive Psychotherapy, 29(3), 367–372. 10.1017/s1352465801003095

[jts23164-bib-0031] Hackmann, A. , Ehlers, A. , Speckens, A. , & Clark, D. M. (2004). Characteristics and content of intrusive memories in PTSD and their changes with treatment. Journal of Traumatic Stress, 17(3), 231–240. 10.1023/B:JOTS.0000029266.88369.fd 15253095

[jts23164-bib-0032] Harvey, A. G. , & Bryant, R. A. (1999). A qualitative investigation of the organization of traumatic memories. British Journal of Clinical Psychology, 38(4), 401–405. 10.1348/014466599162999 10590827

[jts23164-bib-0033] Hellawell, S. J. , & Brewin, C. R. (2002). A comparison of flashbacks and ordinary autobiographical memories of trauma: Cognitive resources and behavioural observations. Behaviour Research and Therapy, 40(10), 1143–1156. 10.1016/s0005-7967(01)00080-8 12375723

[jts23164-bib-0034] Hellawell, S. J. , & Brewin, C. R. (2004). A comparison of flashbacks and ordinary autobiographical memories of trauma: Content and language. Behaviour Research and Therapy, 42(1), 1–12. 10.1016/s0005-7967(03)00088-3 14744519

[jts23164-bib-0035] Hiskey, S. , Luckie, M. , Davies, S. , & Brewin, C. R. (2008a). The emergence of posttraumatic distress in later life: A review. Journal of Geriatric Psychiatry and Neurology, 21(4), 232–241. 10.1177/0891988708324937 19017780

[jts23164-bib-0036] Hiskey, S. , Luckie, M. , Davies, S. , & Brewin, C. R. (2008b). The phenomenology of reactivated trauma memories in older adults: A preliminary study. Aging & Mental Health, 12(4), 494–498. 10.1080/13607860802224367 18791897

[jts23164-bib-0037] Hoppe, J. M. , Wallden, Y. S. E. , Kanstrup, M. , Singh, L. , Agren, T. , Holmes, E. A. , & Moulds, M. L. (2022). Hotspots in the immediate aftermath of trauma—Mental imagery of worst moments highlighting time, space and motion. Consciousness and Cognition, 99, Article 103286. 10.1016/j.concog.2022.103286 35220032

[jts23164-bib-0038] Horowitz, M. J. (1976). Stress response syndromes. Jason Aronson.

[jts23164-bib-0039] Hyland, P. , Shevlin, M. , & Brewin, C. R. (2023). The memory and identity theory of *ICD‐11* complex posttraumatic stress disorder. Psychological Review, 130(4), 1044–1065. 10.1037/rev0000418 37338431

[jts23164-bib-0040] Hyland, P. , Shevlin, M. , Martsenkovskyi, D. , Ben‐Ezra, M. , & Brewin, C. R. (2024). Testing predictions from the memory and identity theory of *ICD‐11* complex posttraumatic stress disorder: Measurement development and initial findings. Journal of Anxiety Disorders, 105, Article 102898. 10.1016/j.janxdis.2024.102898 38991292

[jts23164-bib-0041] Jacobs, W. J. , & Nadel, L. (1985). Stress‐induced recovery of fears and phobias. Psychological Review, 92(4), 512–531. 10.1037//0033-295x.92.4.512 3903814

[jts23164-bib-0042] Janoff‐Bulman, R. (1992). Shattered assumptions: Towards a new psychology of trauma. Free Press.

[jts23164-bib-0043] Johnson, M. K. (2006). Memory and reality. American Psychologist, 61(8), 760–771. 10.1037/0003-066x.61.8.760 17115808

[jts23164-bib-0044] Kim, J. J. , & Diamond, D. M. (2002). The stressed hippocampus, synaptic plasticity and lost memories. Nature Reviews Neuroscience, 3(6), 453–462. 10.1038/nrn849 12042880

[jts23164-bib-0045] Kleim, B. , Bingisser, M.‐B. , Westphal, M. , & Bingisser, R. (2015). Frozen moments: Flashback memories of critical incidents in emergency personnel. Brain and Behavior, 5(7), Article e00325. 10.1002/brb3.325 26221567 PMC4511283

[jts23164-bib-0046] Kleim, B. , Ehlers, A. , & Glucksman, E. (2007). Early predictors of chronic post‐traumatic stress disorder in assault survivors. Psychological Medicine, 37(10), 1457–1467. 10.1017/s0033291707001006 17588274 PMC2829994

[jts23164-bib-0047] Kleim, B. , Graham, B. , Bryant, R. A. , & Ehlers, A. (2013). Capturing intrusive reexperiencing in trauma survivors’ daily lives using ecological momentary assessment. Journal of Abnormal Psychology, 122(4), 998–1009. 10.1037/a0034957 24364602 PMC3906879

[jts23164-bib-0048] Kroes, M. C. W. , Whalley, M. G. , Rugg, M. D. , & Brewin, C. R. (2011). Association between flashbacks and structural brain abnormalities in posttraumatic stress disorder. European Psychiatry, 26(8), 525–531. 10.1016/j.eurpsy.2011.03.002 21592738

[jts23164-bib-0049] Kvavilashvili, L. (2014). Solving the mystery of intrusive flashbacks in posttraumatic stress disorder: Comment on Brewin (2014). Psychological Bulletin, 140(1), 98–104. 10.1037/a0034677 24364748

[jts23164-bib-0050] Lindsay, D. S. , & Read, J. D. (1995). “Memory work” and recovered memories of childhood sexual abuse: Scientific evidence and public, professional, and personal issues. Psychology Public Policy and Law, 1(4), 846–908. 10.1037/1076-8971.1.4.846

[jts23164-bib-0051] Macdonald, B. , Salomons, T. V. , Meteyard, L. , & Whalley, M. G. (2018). Prevalence of pain flashbacks in posttraumatic stress disorder arising from exposure to multiple traumas or childhood traumatization. Canadian Journal of Pain, 2(1), 48–56. 10.1080/24740527.2018.1435994 35005365 PMC8730607

[jts23164-bib-0052] Massazza, A. , Joffe, H. , & Brewin, C. R. (2021). Intrusive memories following disaster: Relationship with peritraumatic responses and later affect. Journal of Abnormal Psychology, 130(7), 727–735. 10.1037/abn0000694 34435809

[jts23164-bib-0053] Massazza, A. , Joffe, H. , Hyland, P. , & Brewin, C. R. (2021). The structure of peritraumatic reactions and their relationship with PTSD among disaster survivors. Journal of Abnormal Psychology, 130(3), 248–259. 10.1037/abn0000663 33539115

[jts23164-bib-0054] McCarthy‐Jones, S. , & Longden, E. (2015). Auditory verbal hallucinations in schizophrenia and post‐traumatic stress disorder: Common phenomenology, common cause, common interventions? Frontiers in Psychology, 6, Article 1071. 10.3389/fpsyg.2015.01071 26283997 PMC4517448

[jts23164-bib-0055] McNally, R. J. (2003). Remembering trauma. Harvard University Press.

[jts23164-bib-0056] Metcalfe, J. , Brezler, J. C. , McNamara, J. , Maletta, G. , & Vuorre, M. (2019). Memory, stress, and the hippocampal hypothesis: Firefighters’ recollections of the fireground. Hippocampus, 29(12), 1141–1149. 10.1002/hipo.23128 31254433

[jts23164-bib-0057] Michael, T. , Ehlers, A. , Halligan, S. L. , & Clark, D. M. (2005). Unwanted memories of assault: What intrusion characteristics are associated with PTSD? Behaviour Research and Therapy, 43(5), 613–628. 10.1016/j.brat.2004.04.006 15865916

[jts23164-bib-0058] Morgan, C. A. , Hazlett, G. , Doran, A. , Garrett, S. , Hoyt, G. , Thomas, P. , Baranoski, M. , & Southwick, S. M. (2004). Accuracy of eyewitness memory for persons encountered during exposure to highly intense stress. International Journal of Law and Psychiatry, 27(3), 265–279. 10.1016/j.ijlp.2004.03.004 15177994

[jts23164-bib-0059] Mota, N. , Tsai, J. , Kirwin, P. D. , Harpaz‐Rotem, I. , Krystal, J. H. , Southwick, S. M. , & Pietrzak, R. H. (2016). Late‐life exacerbation of PTSD symptoms in US veterans: Results from the National Health and Resilience in Veterans Study. Journal of Clinical Psychiatry, 77(3), 348–354. 10.4088/JCP.15m10101 27046308

[jts23164-bib-0060] Nijdam, M. J. , Baas, M. A. M. , Olff, M. , & Gersons, B. P. R. (2013). Hotspots in trauma memories and their relationship to successful trauma‐focused psychotherapy: A pilot study. Journal of Traumatic Stress, 26(1), 38–44. 10.1002/jts.21771 23315999

[jts23164-bib-0061] Pearson, M. , Brewin, C. R. , Rhodes, J. , & McCarron, G. (2008). Frequency and nature of rumination in chronic depression: A preliminary study. Cognitive Behaviour Therapy, 37(3), 160–168. 10.1080/16506070801919224 18608314

[jts23164-bib-0062] Priebe, K. , Kleindienst, N. , Zimmer, J. , Koudela, S. , Ebner‐Priemer, U. , & Bohus, M. (2013). Frequency of intrusions and flashbacks in patients with posttraumatic stress disorder related to childhood sexual abuse: An electronic diary study. Psychological Assessment, 25(4), 1370–1376. 10.1037/a0033816 23876157

[jts23164-bib-0063] O'Kearney, R. , Speyer, J. , & Kenardy, J. (2007). Children's narrative memory for accidents and their post‐traumatic distress. Applied Cognitive Psychology, 21(7), 821–838. 10.1002/acp.1294

[jts23164-bib-0064] Recovered Memory Archive . (n.d.) https://www.recoveredmemory.org/case‐archive

[jts23164-bib-0065] Redican, E. , Nolan, E. , Hyland, P. , Cloitre, M. , McBride, O. , Karatzias, T. , Murphy, J. , & Shevlin, M. (2021). A systematic literature review of factor analytic and mixture models of *ICD‐11* PTSD and CPTSD using the International Trauma Questionnaire. Journal of Anxiety Disorders, 79, Article 102381. 10.1016/j.janxdis.2021.102381 33714868

[jts23164-bib-0066] Reynolds, M. , & Brewin, C. R. (1999). Intrusive memories in depression and posttraumatic stress disorder. Behaviour Research and Therapy, 37(3), 201–215. 10.1016/s0005-7967(98)00132-6 10087639

[jts23164-bib-0067] Rubin, D. C. , Berntsen, D. , & Bohni, M. K. (2008). Memory‐based model of posttraumatic stress disorder: Evaluating basic assumptions underlying the PTSD diagnosis. Psychological Review, 115(4), 985–1011. 10.1037/a0013397 18954211 PMC2762652

[jts23164-bib-0068] Rubin, D. C. , Berntsen, D. , Ogle, C. M. , Deffler, S. A. , & Beckham, J. C. (2016). Scientific evidence versus outdated beliefs: A response to Brewin (2016). Journal of Abnormal Psychology, 125(7), 1018–1021. 10.1037/abn0000211 27732030 PMC5063074

[jts23164-bib-0069] Rubin, D. C. , Deffler, S. A. , Ogle, C. M. , Dowell, N. M. , Graesser, A. C. , & Beckham, J. C. (2016). Participant, rater, and computer measures of coherence in posttraumatic stress disorder. Journal of Abnormal Psychology, 125(1), 11–25. 10.1037/abn0000126 26523945 PMC4701605

[jts23164-bib-0070] Rusch, H. L. , Robinson, J. , Yun, S. , Osier, N. D. , Martin, C. , Brewin, C. R. , & Gill, J. M. (2019). Gene expression differences in PTSD are uniquely related to the intrusion symptom cluster: A transcriptome‐wide analysis in military service members. Brain Behavior and Immunity, 80, 904–908. 10.1016/j.bbi.2019.04.039 31039430 PMC6752960

[jts23164-bib-0071] Sachs‐Ericsson, N. , Joiner, T. E. , Cougle, J. R. , Stanley, I. H. , & Sheffler, J. L. (2016). Combat exposure in early adulthood interacts with recent stressors to predict PTSD in aging male veterans. Gerontologist, 56(1), 82–91. 10.1093/geront/gnv036 26220414

[jts23164-bib-0072] Sandi, C. , & Pinelo‐Nava, M. T. (2007). Stress and memory: Behavioral effects and neurobiological mechanisms. Neural Plasticity, 2007, Article 78970. 10.1155/2007/78970 18060012 PMC1950232

[jts23164-bib-0073] Shin, L. M. , McNally, R. J. , Kosslyn, S. M. , Thompson, W. L. , Rauch, S. L. , Alpert, N. M. , Metzger, L. J. , Lasko, N. B. , Orr, S. P. , & Pitman, R. K. (1999). Regional cerebral blood flow during script‐driven imagery in childhood sexual abuse‐related PTSD: A PET investigation. American Journal of Psychiatry, 156(4), 575–584. 10.1176/ajp.156.4.575 10200737

[jts23164-bib-0074] Shobe, K. K. , & Kihlstrom, J. F. (1997). Is traumatic memory special? Current Directions in Psychological Science, 6(3), 70–74. 10.1111/1467-8721.ep11512658

[jts23164-bib-0075] Speckens, A. E. M. , Ehlers, A. , Hackmann, A. , & Clark, D. M. (2006). Changes in intrusive memories associated with imaginal reliving in posttraumatic stress disorder. Journal of Anxiety Disorders, 20(3), 328–341. 10.1016/j.janxdis.2005.02.004 16564436

[jts23164-bib-0076] Speckens, A. E. M. , Ehlers, A. , Hackmann, A. , Ruths, F. A. , & Clark, D. M. (2007). Intrusive memories and rumination in patients with post‐traumatic stress disorder: A phenomenological comparison. Memory, 15(3), 249–257. 10.1080/09658210701256449 17454662

[jts23164-bib-0077] Taylor, A. , Zajac, R. , Takarangi, M. K. T. , & Garry, M. (2022). Evidence from the trauma–film paradigm that traumatic and nontraumatic memories are statistically equivalent on coherence. Clinical Psychological Science, 10(3), 417–429. 10.1177/21677026211053312

[jts23164-bib-0078] Tulving, E. (1985). Memory and consciousness. Canadian Psychology/Psychologie canadienne, 26(1), 1–12. 10.1037/h0080017

[jts23164-bib-0079] Van der Kolk, B. A. , Brown, P. , & Van der Hart, O. (1989). Pierre Janet on post‐traumatic stress. Journal of Traumatic Stress, 2(4), 365–378. 10.1002/jts.2490020403

[jts23164-bib-0080] Whalley, M. G. , Kroes, M. C. W. , Huntley, Z. , Rugg, M. D. , Davis, S. W. , & Brewin, C. R. (2013). An fMRI investigation of posttraumatic flashbacks. Brain and Cognition, 81(1), 151–159. 10.1016/j.bandc.2012.10.002 23207576 PMC3549493

[jts23164-bib-0081] Wolf, E. J. , Hawn, S. E. , Sullivan, D. R. , Miller, M. W. , Sanborn, V. , Brown, E. , Neale, Z. , Fein‐Schaffer, D. , Zhao, X. , Logue, M. W. , Fortier, C. B. , McGlinchey, R. E. , & Milberg, W. P. (2023). Neurobiological and genetic correlates of the dissociative subtype of posttraumatic stress disorder. Journal of Psychopathology and Clinical Science, 132(4), 409–427. 10.1037/abn0000795 37023279 PMC10286858

[jts23164-bib-0082] World Health Organization . (2019). International statistical classification of diseases and related health problems (11th ed.). https://icd.who.int/ 10.2471/BLT.25.294650 PMC13531092 42683092

